# Engagement Features in Physical Activity Smartphone Apps: Focus Group Study With Sedentary People

**DOI:** 10.2196/20460

**Published:** 2020-11-16

**Authors:** Marco D'Addario, Dario Baretta, Francesco Zanatta, Andrea Greco, Patrizia Steca

**Affiliations:** 1 Department of Psychology University of Milan-Bicocca Milan Italy; 2 Department of Human and Social Sciences University of Bergamo Bergamo Italy

**Keywords:** physical activity, smartphone app, engagement, co-design, thematic analysis, mobile phone

## Abstract

**Background:**

Engagement with physical activity mobile apps has been reported to be a core precondition for their effectiveness in digital behavior change interventions. However, to date, little attention has been paid to understanding the perspectives, needs, expectations, and experiences of potential users with physical activity mobile apps.

**Objective:**

The aim of this study was to investigate the features that are judged to be important for engagement with a physical activity mobile app and the reasons for their importance.

**Methods:**

A qualitative focus-group methodology with elements of co-design was adopted in this study. Participants reporting sedentary lifestyles and willingness to improve their physical activity behavior through mobile technology were recruited. The focus group sessions consisted of 13 participants (8 men and 5 women, mean [SD] age 41.9 [7.1] years). Two researchers conducted the data analysis independently by using the inductive thematic approach.

**Results:**

Four main themes emerged in relation to the research question and were named as follows: “physical activity participation motives,” “autonomy and self-regulation,” “need for relatedness,” and “smart.” Additionally, 2 subthemes originated from “physical activity participation motives” (ie, “medical guidance” and “weight loss and fitness for health”) and “smart” (ie, “action planning” and “adaptable and tailored”).

**Conclusions:**

Features enhancing autonomy and self-regulation and positively affecting health and physical well-being as well as the need for relatedness, adaptability, and flexibility should be considered as core elements in the engagement of potential users with physical activity mobile apps. The emerged findings may orient future research and interventions aiming to foster engagement of potential users with physical activity apps.

## Introduction

### Background

Regular physical activity is widely recognized as a protective factor against cardiovascular diseases, diabetes, obesity, and some forms of cancer [1-3]. Nevertheless, a third of the adults worldwide are insufficiently physically active [4]; as a consequence, promoting effective interventions targeting physical activity is a priority. Behavior change interventions that increase physical activity have been shown to be effective [5], and diverse findings supporting their cost-effectiveness have been reported [6]. However, most of these interventions have been delivered with a face-to-face methodology, which is considered, for intrinsic reasons, unsuitable for targeting large populations and supporting behavior change in ecological settings.

Mobile technology provides a valid tool to foster interventions [7,8] and to reach large populations [9]. Nevertheless, little evidence has been provided so far for the modest effect of web-based and mobile-based interventions on physical activity [9-11]. A recent systematic review and meta-analysis has aimed to determine the role of smartphone apps in increasing objectively measured physical activity in adults and it provided modest evidence supporting short-term effectiveness (eg, up to 3 months) [12]. In this regard, engagement was shown to be a fundamental precondition for digital behavior change interventions (eg, physical activity smartphone apps) and their effectiveness [13]. Nevertheless, digital interventions were shown to have low engagement in different contexts, including controlled trials [10,14] and in relation to health app usage in real life settings [15]. Specifically, the lack of desired features and abandoning health goals were reported as reasons for low engagement and subsequent dropout [16]. Conversely, other studies have suggested higher engagement with health smartphone apps to be potentially supported by interactive features such as social and professional support, self-monitoring, and feedback [17-19]. However, in spite of recommendations [20,21], little attention has been paid to understanding users’ perspectives and preferences of these features and implementing them in app functionalities in order to sustain users’ engagement with behavior change and health-related goals. Consistently to this point, a recent systematic review unveiled that personal factors and features of the device play a role in influencing participants’ motivation to engage with mobile apps promoting physical activity [22]. It was noted that, among the personal factors, prior experience with and rationales for using health apps influenced users’ motivation. Moreover, mobile health components were found to potentially facilitate changing users’ ways of thinking and self-awareness in relation to physical activity. Indeed, the monitoring features of the apps gave the users opportunities to reflect, which led them to include additional physical activity in their routines. Furthermore, social features, prompts, goal setting, and gamification were noted as determinants of a better experience of mobile health in physical activity, suggesting higher engagement with app use. Thus, ease of use, personalized features, and the possibility to customize the app were shown as relevant factors too. Another recent qualitative study synthesized what is known about influences on the uptake of and engagement with health smartphone apps, suggesting that factors such as physical and psychological capability, physical and social opportunity, and motivation are pivotal and deserve further investigation [23]. Especially for engagement, app instructions, health and well-being information, and visual or numerical summary of progress were considered essential. In addition, self-monitoring, established routines, and safety netting have been taken into account as pivotal features supporting behavioral regulation and consequent engagement with the app.

For these purposes and according to previous observations, a mixed-methods research design with a specific focus on qualitative methodologies (eg, focus groups, interview with open-ended questions, think-aloud studies) may be preferred [21,24,25]. Qualitative methodologies may indeed benefit from various techniques. One in particular that is gaining attention from mobile health researchers is co-design. The term “co-design” refers to the creative collaboration between researchers and end users and to their involvement in the design development process, where the valuable role of the latter relies on their position as “experts of their experience” [26]. *Co-designing*, for instance, a digital intervention in cooperation with potential users encompasses advantages, including better idea generation and better fit between users and the product, higher quality, and more effective products as well as more efficiency in project management [27].

### Objective

The aim of this study was to elicit the preferences and perspectives of sedentary potential users toward features of a physical activity mobile app. Hence, we intend to elucidate why specific functionalities and features are judged to be important in the design process of a physical activity mobile app that contributes to increasing potential users’ engagement.

## Methods

### Study Design

This study adopted a focus group methodology with elements of co-design. This study design aimed to address users’ perspectives and expectations in relation to a physical activity mobile app. The cocreation of a fit-for-purpose digital product designed with and around the users represents the real added value of this methodology. Co-design activities allow users to express their creativity and contribute to the development process as experts of their own experiences [26]. The project design, procedures, and informed consent form were approved by the ethics committee of the University of Milan-Bicocca.

### Study Participants

The participants were recruited according to the following eligibility criteria: (1) age between 30 years and 50 years, (2) no preexisting health conditions that would impede physical activity, (3) no clinical conditions related to physical inactivity (eg, obesity, diabetes), (4) insufficient physical activity screened with the Global Activity Questionnaire [28] and consequently compared to physical activity recommended guidelines (ie, 150 minutes of moderate physical activity or 75 minutes of vigorous physical activity per week), and (5) interest in increasing physical activity behavior through mobile technologies. No monetary compensation for participation was provided.

### Sampling

The aim of the focus group and the eligibility criteria were specified in the recruitment materials (social media, snowball sampling methods, and posters placed on the university campus). Recruitment stopped when no further relevant insights of participants’ experiences and novel themes emerged from the focus groups [29].

### Co-Design Materials

While the materials were being developed, design features were primarily defined in order to fulfil the most relevant physical activity participation motives and to implement the behavior change techniques (eg, action planning, goal setting, problem solving, self-monitoring) [30]. Additional features that are expected to be associated with engagement (ie, rewards, reminders, social support) [18] were also included as stimuli. A co-design pack was created according to a preliminary revision of the literature. The selected functions and features presented to participants differed between the 2 focus groups. In the first focus group, the features were written on post-its and referred to the behavior change techniques recognized as the most effective in physical activity promotion [5] and to the different motives associated with physical activity [31,32]. In the second focus group, the features were presented as images and referred to those mainly included in the publicly available physical activity apps [33]. Changes of materials between the first and the second focus group were made with the aim to provide the participants with a more engaging and realistic co-design activity, since interacting with images may be more stimulating than post-its. Although the creative activities formed a large proportion of the work, co-design materials and the related creative tasks were exclusively considered as stimuli for participants in focus group conduction and were not included in the data analysis phase.

### Procedure

Participants were invited to the University of Milan-Bicocca. Before starting the focus group session, participants were given the information sheet describing the nature of the study. They subsequently signed the informed consent form. As the first focus group activity, participants introduced themselves and shared their previous personal experiences with physical activity and with mobile apps. Subsequently, a folder with materials for co-design was provided. Participants were asked to design their own physical activity app according to their preferences for specific functionalities and features. According to the user-centered methodology and the iterative nature of the co-design process, materials and the track for the following co-design sessions were consequently adapted in order to better elicit users’ views about what specific functionalities are expected to be important and why. The first session consisted of evaluating and designing potential physical activity app features (functionalities and features were written on post-its given to participants at the beginning of the focus group session) according to different topics (ie, feedback and monitoring, goal setting and planning, challenges and social features, problem solving). A brief discussion was then conducted when each topic was completed. In the latter session, participants were asked to cut out images of features printed on a paper sheet and paste them on a printed smartphone frame according to their preferences. Once the whole activity was accomplished, a general discussion was conducted. In both co-design sessions, participants were also provided with blank post-its to suggest any further functionalities they would like to see implemented in a physical activity app. Changes to the co-design task relied on the aim to characterize the activity with more concrete stimuli and to stress the creative act of cocreating the product. Therefore, this did not influence the scope of focus group sessions (ie, elicit potential users’ views and preferences for design features) but, rather, provided an opportunity for a better expression of participants’ views and perspectives.

### Data Analysis

The focus group sessions were audio-recorded and transcribed verbatim. The transcripts were qualitatively analyzed using inductive thematic analysis [34]. Thematic analysis consists of 6 phases: (1) familiarizing with the data, (2) generating initial codes, (3) searching for themes, (4) reviewing themes, (5) defining and naming themes, and (6) producing the report. Given the exploratory nature of the thematic analysis methodology, it was expected that diverse themes with different contents might emerge [34]. For this reason, the themes might not be strictly connected to engagement. Therefore, any theme that emerged and that was not necessarily related to engagement was accepted as an informative finding. Data and repeated patterns that were considered pertinent to the aim of the study were coded by 2 researchers working independently. New inductive codes were labelled as they were identified during the coding process and the results of the coding were iteratively revised. The next stage involved searching for themes; both researchers reviewed their own generated codes one by one, organizing the findings in order to combine the different codes that have been considered focusing on the same aspect. Finally, the codes generated by the 2 researchers were compared and subsequently organized into themes. Discussion within the entire research group was conducted in order to reach a consensus on the final themes. Once themes were defined and named, examples of transcripts were selected to corroborate themes on the basis of their representativeness and relevance. Data were analyzed in their original language in order to preserve the participants’ original meanings, while coding and themes were formulated in English only. The selected examples of transcripts were translated into English for illustration purposes.

## Results

### Overview of the Findings

Two focus groups were formed (N=13, 8 men and 5 women). The mean (SD) age of the participants was 41.9 (7.1) years. All the participants reported doing less than 120 minutes of light-to-moderate physical activity per week; 4 participants reported having previously used smartphone apps to support physical activity.

Four themes were developed in relation to the research question and were named as follows: “physical activity participation motives,” “autonomy and self-regulation,” “need for relatedness,” and “smart.” Two subthemes were developed in relation to the “physical activity participation motives” theme: “medical guidance” and “weight loss and fitness.” Additionally, 2 subthemes originated from the “smart” theme: “action planning” and “adaptable and tailored” (see Table 1 for a brief description of the themes and subthemes).

**Table 1:** Summary of the themes and subthemes that emerged from the thematic analysis.

Themes and subthemes		Description
**Physical activity participation motives**		The intention to do physical activity is more related to health and well-being or duty motives, rather than intrinsic motivation or pleasure.
	Medical guidance	A physical activity app should contain features that include medical support, providing feedback about physiological parameters related to health.
	Weight loss and fitness for health	A physical activity app should contain features that contribute to physical benefits and cardiovascular fitness.
Autonomy and self-regulation		Physical activity app design should include features (eg, monitoring, goal setting, feedback) that contribute to increasing self-regulation and autonomous behaviors.
Need for relatedness		It is crucial that a physical activity app contains features that leverage the motivational component of social support and promote privacy, safety, and comparison with oneself.
**Smart**		Subjects ask for a flexible physical activity mobile app that provides context-aware suggestions and goal-oriented feedback.
	Action planning	Physical activity app design should include activity suggestions in relation to users’ personal goals and flexible programs.
	Adaptable and tailored	Potential users would prefer a customized physical activity mobile app that can deliver tailored suggestions and that is built on their own characteristics and motivations.

### Physical Activity Participation Motives

Participants highlighted the importance of using a physical activity app focusing on participation motives. Most of them reported that their intention to practice physical activity did not originate from intrinsic motivation or pleasure, but from health and well-being–related or duty motives.

…I consider physical activity as a responsibility to feel healthy, rather than a pleasure. It is an effort that I make to feel better.

Male #1

…For some people, fitness is a pleasure. Personally, I would exercise because it would make me healthy, not because I’d like it.

Male #2

Specifically, physical activity participation motives can be attributable to the desire for medical guidance and a specific focus on weight loss and fitness.

#### Subtheme 1 of Physical Activity Participation Motives: Medical Guidance

Positive opinions about the opportunity to be informed and supported by medical sources (eg, medical doctors or devices) were expressed. This aspect would contribute to making the app more credible, reliable, and trustworthy. Moreover, thanks to the presence of a medical perspective, participants expected the app to be able to provide feedback about the physiological parameters strictly related to health.

…Physical activity should be supported and monitored by a sports doctor, and after an exercise electrocardiogram… It could be dangerous otherwise!

Male #3

…I would like to be supported by a virtual personal trainer, but I need something more such as a doctor, an expert who gives reliable and evidence-based recommendations.

Male #4

#### Subtheme 2 of Physical Activity Participation Motives: Weight Loss and Fitness for Health

Focus group participants remarked the importance of physical activity for weight loss and fitness, thereby contributing to physical benefits and cardiovascular health.

…My eating habits are important but my physical well-being is also a fundamental aim for me: to get my fitness back, to control my body weight and my heart […] I always imagine an app matching with my main aim, which is weight loss, blood pressure and heart control. I imagine an app as an instrument supporting physical well-being.

Male #5

…I am interested because my hope is to lose weight. I have perennial fight with my weight…”

Female #2

### Autonomy and Self-Regulation

Most of the participants reported monitoring, goal setting, and feedback as the key components for a physical activity app design. These features would help increase not only self-regulation in physical activity but also autonomous behaviors.

…It must be something [app] that helps me to reach my goal. It should be a calculator of what I did and what I should have done…

Male #5

…I consider information like how far and how long I have run, the distance and other similar data that I consider the starting point. These may then help to add further elements that an app should offer.

Female #1

…you receive a notification on your smartphone like “you are worsening, your conditions are decreasing”

Male #1

In particular, regarding monitoring and feedback features, most participants said that they were interested in having records and statistics about their itineraries.

…I would like my physical activity to be monitored and analyzed with charts and stats. I would prefer to see daily, weekly, and monthly statistics.

Male #2

…I would like the app to track my paths. It could motivate me to do more, improving my results. Also, I imagine an app providing an archive of previous paths.

Male #4

### Need for Relatedness

Focus group participants expressed social support as a fundamental component that could help to create higher levels of daily commitment, to make exercise a more enjoyable activity, as well as to increase motivation and overcome laziness.

…I should find someone who motivates my goal setting. Not daily goals, but goals helping me to use the app regularly […] It would push me to share with others, to be more active…

Female #2

…If you have an appointment, then you can’t quit.

Female #3

…I have always been very lazy. Laziness leads me to be idle when I’m alone. So, it would be better if there was someone with me.

Female #4

…You have to find someone at your own pace.

Male #2

Although peer support was shown to be a core element, most of the participants expressed avoidance to social comparison, exhibitionism, and competition, and preferred privacy, safety, and comparison with oneself.

…I almost never share my private life […] I don’t think it is interesting to know or to let others know what I usually do.

Female #1

…It could be dangerous to let you know what I do, or to let me know what you do every day.

Male #3

…I would exercise for personal motives and not for competing […] Everything I do is for a comparison with myself: I like seeing my own improvements.

Male #1

Furthermore, the possibility to interact with a virtual personal trainer who is aware of the personal motivation, abilities, and preferences was reported as a core element.

…I would like to rub the smartphone to bring up a personal trainer […] I do not perceive the human component. I would need the relationship with a personal trainer, with someone able to show the exercises right in front of you.

Female #5

…I would need an app like a personal trainer who monitors me […] Someone who supports me.

Female #2

…It should be something that gives tailored hints, knowing one’s motivations… like a parent who knows you, who loves you, who says what to do.

Male #3

…and then, I would like something like Siri that asks to you before you start: “How do you feel today?” “Today, I don’t want to put in too much effort” “Ok, I will choose this path for you, then!

Female #3

### Smart

Participants imagined a physical activity mobile app presenting flexibility and adaptability toward individual circumstances and motivations. For this purpose, the app should provide context-aware suggestions, goal-oriented feedback, and action-oriented planning for a tailored support in physical activity.

#### Subtheme 1 of Smart: Action Planning

Participants expressed the preference to use a physical activity app that consisted of features that suggest activities in relation to personal goals and flexible programs.

…I would like the app to propose a training. An app suggesting a training according to my goal.

Male #6

…an app helping me to identify a lighter training instead of forcing me when I am particularly tired…

Female #3

…I would need an app suggesting a path, for example, “Today, you have one hour available and you could choose this path. There is a park nearby.”

Female #2

…an app identifying paths or outdoor gyms. So, for example, when I don’t know where to go to run it could tell me, “These are some paths you might be interested in.”

Female #1

#### Subtheme 2 of Smart: Adaptable and Tailored

Most of the participants believed that a physical activity app should be customized to users’ characteristics and motivations and should also be able to read and detect behaviors and needs and deliver tailored suggestions.

…to customize it as much as possible, it should be tailored […] If the app knows that you have high blood pressure, it will not suggest you go running for 3 hours.

Female #1

…It should be as complete as possible. It should not be based on only one parameter, but rather on different characteristics […] It should be customized, for example, according to one’s psychological and physical condition at the specific moment of the day.

Female #2

…Based on the data on your goals you fill in when registering, the app should modulate the difficulty and give some short term results to achieve your goals.

Male #7

…Everything needs to be flexible considering that I am flexible.

Male #3

## Discussion

### Principal Results

In this study, we investigated the potential features of a physical activity app that are judged to be important for engagement by sedentary potential users and the reasons for their importance. A focus group methodology with elements of co-design was adopted in order to involve participants who reflected about the app features and researchers who characterized the design process. The findings of this study suggest that features that enhance users’ autonomy and self-regulation and those that focus on the impact physical activity has on health and physical well-being were considered to be relevant for engagement. The need for relatedness was considered as a trigger for exercise if it adopts a supportive and motivational style. For this purpose, both a virtual personal trainer with human qualities embedded into the app and opportunities to develop a network of physical activity peers were judged as relevant features. A different aspect noticed in this study was that participants seemed to give importance to the private dimension of exercising and, thus, expressed a preference for self-comparison, privacy, and safety rather than competition, exhibitionism, and social comparison. Finally, participants believed that a smart, tailored interaction between user and app would foster a more effective engagement by providing an adaptable flexible approach for users to achieve personal goals.

### Comparison With Prior Work

The intentions and motives related to exercise seemed to be mostly oriented toward health and physical well-being concerns, which were partially coherent with those reported in a previous research [19]. In the previous study [19], specific features focusing on fitness, nutrition, and weigh loss were listed as the main reasons for engaging in physical activity. In addition, a preference for particular suggestions and the possibility to track relevant health-related parameters was indicated. As for what emerged in this research, improving cardiovascular fitness and weight loss and monitoring of health-related indicators were repeatedly reported as the primary motivations for being more active and healthier. To address this request, physical activity apps should take into account a wider and more integrated approach for physical activity by involving medical guidance to help users to achieve and monitor their health-related goals (eg, weight control, aerobic improvements). For these purposes, new emerging mobile technology such as self-tracking devices may provide more reliable, integrated information about physical activity behavior and its outcomes (eg, indicators of weight loss, index of fitness, heart rate).

The preferences of the potential users for autonomy and self-regulation features were consistent with those reported in a previous research [19,35] and corroborated the results from a recent review [18], suggesting that behavior change techniques such as goal setting, feedback, and self-monitoring are associated with higher engagement. Specifically, these features allow participants to monitor their improvements, highlighting any potential discrepancy between their physical activity behavior and their goal. Moreover, self-regulation features (ie, goal setting, behavior monitoring, receiving feedback) have been shown to be effective in increasing physical activity [36]. Similar convergent empirical evidence has clearly validated the idea that considering self-regulation features in a physical activity app allows users to self-organize their experience and behavior, thereby fostering their autonomy in exercising [19]. Furthermore, they deserve to be considered as the core component of physical activity apps, as they have already been expected to increase engagement [17,18], be appreciated by users [37], and be associated with physical activity intervention effectiveness [5,38]. It is noteworthy that although commercial physical activity apps often implement such features [39], this might be done in a more theory-driven way as highlighted for goal setting in a recent review and content analysis of commercial apps [40].

The need for relatedness was perceived by potential physical activity app users as a core element for increasing their engagement with the app. Previous research has shown social support features not to be as relevant as the self-regulation feature [41]. In contrast, a recent study [19] supported the motivational aspect of social interactions as an element supporting and fostering higher levels of commitment. Indeed, it was unveiled that although varying types and levels of needs for relatedness with others were expressed, connection with peers and coaching features embedded into the app play a promoting role, thereby increasing motivation and making physical activity more enjoyable. Consistently, the findings of this study show that human interactions with peers and virtual personal trainers are judged to be a trigger for being more active and overcoming laziness, as well as a fundamental feature in support and consequent dedication. Indeed, features connecting the user with a virtual coach or a group of people with similar goals were expected to be important for engagement because of the formation of a shared commitment and the opportunity to be emotionally supported. The findings in this study are consistent with those reported in a previous study [42], suggesting that working alliance and the desire to continue using a digital behavior change intervention to promote physical activity are increased by the support provided by a computer interface with human-relational skills (eg, empathy, social dialogue). Furthermore, a scoping review of web-based interventions showed supportive virtual coaches to be a potentially valuable remedy for low adherence to digital interventions [43]. In accordance with previous research targeting different behaviors [18,37], potential users of physical activity apps are reluctant to share information with social networks about their physical activity. Indeed, users perceived physical activity behavior change as a personal path and did not see merit in showing it to other users. This avoidance of competition and social comparison is consistent with that reported in a previous study [19], as competence and competition were listed among the less relevant motives for practicing physical activity and social comparison was revealed to be one of the less-liked behavior change techniques. Specifically, the latter was also considered as an obstacle for motivation to exercise due to potential exposure to others’ negative judgments. Furthermore, participants definitely rejected the idea of thinking about physical activity from a competitive perspective, probably because any source of social comparison or competition with more successful users might discourage them, thereby constituting an additional stressful element.

Finally, participants imagined a smart, tailored physical activity app that is customized to “understand” the users’ preferences and expectations and consequently support them as and when required. For example, action planning strategies based on the user’s timetable and suggestions tailored to the user’s level of progress toward a specific goal were expected to be more engaging and effective. The findings in this study corroborate previous observations, indicating that providing tailored physical activity plans based on personal goals and current progress would help users implement their engagement and intentions to exercise [19]. A similar need for a tailored approach was also found in a previous research investigating users’ preferences for design features related to smartphone apps for drinking reduction and smoking cessation [18]. Additionally, participants emphasized their interest in a flexible physical activity app that adapts the intervention content to the specific context and variations in the users’ motivational and emotional states in real time. This suggestion is in line with recent emerging research that has focused on the development of just-in-time adaptive interventions [44] and tailoring physical activity interventions on motivational aspects such as self-efficacy beliefs [45,46].

### Limitations

The limitations of this study include the design of the study: a nonlongitudinal design could have limited the long-term investigation of the influence of the features emerged in users’ engagement. For this purpose, further qualitative studies may be conducted comprising a follow-up aimed to evaluate the possible effects on users’ engagement after a period of tailored smartphone app usage. Another limitation of the study is the narrow age range of participants. Actually, the decision to only include adults aged 30-50 years was based on the belief that this would have improved the quality of the findings. Perski et al [18] reported that engagement with digital behavior change interventions is influenced by age (showing a trend toward a positive association between engagement and older age). However, as the moderating role of age on engagement cannot be easily controlled for in qualitative studies, we preferred to avoid recruiting participants younger than 30 years and older than 50 years in order to prevent collecting data from a heterogeneous sample with varying motivations and subjective experiences. Further investigations may be helpful to explore the role of age. Furthermore, the nature of the focus group may have limited the expression of the participants’ true attitudes because of social desirability bias. Finally, as there can be a difference between what is expected to help engagement and what actually has an influence on engagement, a prospective study including a period of physical activity smartphone app use is recommended. In this way, experiencing the app may lead to understanding whether the features actually have the same impact on engagement as expected.

### Conclusions

The findings in this study provide informative data concerning sedentary potential users’ preferences and perspectives toward physical activity smartphone app features. Consistent with that reported in prior works, improving health and physical well-being were indicated as the primary motivations for exercising, as was the possibility to track relevant health-related parameters with the additional support of medical sources. Similarly, features strictly related to the behavior change techniques (ie, goal setting, feedback, and self-monitoring) were preferred to support autonomy and self-regulation. Furthermore, the preference of tailored and customized features “understanding” user’s expectations and level of progress was shown and this corroborated prior observations. Finally, features that leverage the motivational component of social support and that promote privacy, safety, and self-comparison were shown to be crucial. Nevertheless, they still represent an open question, as contrasting results emerged in prior studies. In order to increase users’ engagement with physical activity smartphone apps and, thus, to improve their effectiveness, the features described here should be taken into account in future design processes and in future research aiming to broaden knowledge on mobile health in relation to physical activity.
